# A Scoring System to Determine the Risk Factors Causing Recurrent Pulmonary Thromboembolism

**DOI:** 10.34172/aim.2023.57

**Published:** 2023-07-01

**Authors:** Mutlu Onur Gucsav, Dursun Tatar

**Affiliations:** ^1^Department of Chest Diseases, University of Health Sciences, Izmir Tepecik Training and Research Hospital, Izmir, Turkey; ^2^Department of Chest Diseases, University of Health Sciences, Dr. Suat Seren Training and Research Hospital for Chest Diseases and Thoracic Surgery, Izmir, Turkey

**Keywords:** Pulmonary embolism, Recurrence, Risk factors, Scoring systems

## Abstract

**Background:**

The risk of recurrence in pulmonary embolism is the highest in the first week after the acute event. Although it decreases over time, it may remain high for months depending on compliance with treatment and the nature of the underlying risk factor. Our study aimed to identify risk factors that lead to recurrence in pulmonary thromboembolism (PTE) patients and establish an easy-to-use scoring system that determines the risk of recurrence after the first embolism.

**Methods:**

We retrospectively evaluated 1452 patients who were diagnosed with acute PTE between 7/1/2014 and 7/1/2019. Demographic data, comorbidities and clinical data of the patients, and risk factors were recorded. The relationship of the examined parameters with recurrent PTE was evaluated.

**Results:**

Diabetes mellitus (DM), hypertension, obesity, and the presence of at least one hereditary risk factor were found to be associated with recurrence. The sensitivity of our score was 66.9%, the specificity was 63.2%, the positive predictive value was 19%, and the negative predictive value was 93.7%. The risk of recurrence in the patients identified as high-risk in the scoring system was 3.47 times higher than those identified as low-risk.

**Conclusion:**

In terms of risk of recurrence, special attention should be paid to patients with diabetes, HT, obesity and any of the hereditary risk factors. Using scoring systems to determine the risk of recurrence will be valuable and interesting as it is easy-to-use, gives quick results and provides quantitative results.

## Introduction

 Pulmonary thromboembolism (PTE) is a disease that occurs when the pulmonary arteries are occluded by thrombus fragments originating from various veins of the body.^1^ It is the 3rd most common cardiovascular disease after myocardial infarction (MI) and stroke.^2^ The increase in the elderly population and new developments in diagnostic methods in the last 20 years have led to an increase in its incidence.^3^

 The risk of recurrence in PTE is the highest in the first week after the acute event. Although it decreases over time, it may remain high for months depending on compliance with treatment and the nature of the underlying risk factor.^1^ It has been shown that the 1-year risk of recurrence rate after discontinuation of treatment is 2.5% in patients with any transient risk factor, and approximately 4.5% in patients without any risk factor.^2^ In studies, different risk factors such as increased body mass index (BMI), older age, presence of thrombophilia, male gender, and cancer were found to be associated with recurrence.^4-7^ Detection of risk factors in PTE patients is of great value in terms of determining the risk of recurrence and the duration of anticoagulation.^5^ However, these individual risk factors may fail to determine the risk of recurrence. Therefore, researchers have proposed various scoring systems to determine the risk of recurrent PTE. The HERDOO2 score (Hyperpigmentation, Edema, or Redness in either leg; D-dimer level ≥ 250 μg/L; Obesity BMI ≥ 30; or Older age, ≥ 65) and the DASH score (D-dimer, Age, Male sex, Hormonal therapy) are the most well-known of these scoring systems.^8,9^ In studies related to recurrence in the literature, risk factors vary depending on the country and patient population of the study. In addition, the HERDOO2 and DASH scores have not found a place in routine practice.

 Our study was designed to establish an easy-to-use scoring system that provides quantitative results in determining the risk factors associated with recurrent PTE and the risk of recurrence after the first PTE attack.

## Materials and Methods

###  Study Design-Population

 In our study, sample size was calculated as pre-hoc using statistical analyses. When the sample ratios were taken as 1:1, the sample size was calculated at 138 patients. We included patients who met the inclusion criteria in the relevant time period, because of the retrospective cohort design of our study and to minimize the margin of error and bias that may arise from sample selection. Patients (n = 2514) diagnosed with acute PTE by thorax computed tomography (CT) angiography and ventilation/perfusion (V/P) scintigraphy method between 7/1/2014 and 7/1/2019 were evaluated retrospectively. Patients who had been diagnosed with chronic PTE, patients who had embolism for non-thrombotic reasons, patients who were under the age of 18, and patients whose data had been lost were excluded from the study. Among the patients included in the study, those who had a PTE attack for the first time (n = 1286) were included in one group, and patients with a previous diagnosis of PTE (n = 166) were included in the other group. The data of the patients in both groups regarding age, gender, BMI, smoking habits, comorbid diseases, presence of accompanying deep vein thrombosis (DVT), risk factors, and clinical stage of the disease and treatment methods were recorded. In light of these data, we aimed to determine the risk factors that predispose the patient to recurrent PTE. Risk factors were selected according to the European Society of Cardiology (ESC) Pulmonary Embolism Diagnosis and Treatment Guidelines. Applicability in the study was difficult due to the large number of risk factors defined for venous thromboembolism (VTE) in this guideline. For this reason, a selection was made among the risk factors in the guideline, taking into account previous publications and our clinical experience. The clinical stage of the patients (massive-submassive-nonmassive) was determined using vital signs, echocardiography (ECHO) findings, and a simplified pulmonaryembolism severity index(s-PESI) score.^2^

###  Statistical Methods

 Statistical Package for the Social Sciences (SPSS, Inc., Chicago IL), version 22 was used for statistical analysis in our study. The Kolmogorov-Smirnov and Shapiro-Wilk tests were used to evaluate the normal distribution of continuous variables. Since the *P* value was < 0.05, these parameters were considered not normally distributed. In the comparison of demographic data, clinical and laboratory findings, Mann-Whitney U test was used for those with non-normal distribution, and independent t-test for those with normal distribution. When it was decided to use independent t-test for normally distributed continuous data, the results of Levene’s test were evaluated for homogeneity of variances and necessary corrections were made. For comparison of categorical parameters, large expected cell counts were evaluated using the chi-square test. Exact test results were used for those with large expected cell counts less than 5 in all chi-square evaluations. For model building, the patients’ demographics and clinical data that may play a role in recurrence in patients with PTE were entered into a univariable regression analysis. When scanning variables for regression, the alpha level was accepted at 0.1. Variables with an alpha level < 0.1 were analyzed with multivariable logistic regression. The assumptions underlying the logistic regression model, including the linearity assumption for quantitative predictors such as BMI, were evaluated. Model suitability and data matches were checked with Hosmer and Lemeshow tests. The results are presented with 95% confidence intervals (CIs). A *P* value < 0.05 was considered statistically significant for all tests.

## Results

 Of the 1452 patients included in the study, 166 (11.4%) were patients with recurrent PTE. Comparison of the two groups in terms of demographic characteristics is given inTable 1. When the two groups were compared in terms of age and gender, no significant difference was found between them (*P* = 0.921 and *P*= 0.515, respectively). However, the BMI of the patients in the recurrent PTE group was significantly higher than the other group (*P*< 0.001). The presence of comorbid disease was significantly more frequent in the recurrent PTE group (*P*< 0.001). Obesity (BMI ≥ 30 kg/m^2^) was the most common comorbid disease in the patients in the recurrent PTE group. Hypertension (HT) was the most common comorbid disease in the group of patients with the first PTE attack. While DVT was observed in 27.7% of recurrent PTE patients, this rate was 29.4% in the group of patients with their first PTE attack. There was no statistically significant difference between the groups (*P*= 0.913). When the patients in the two groups were compared in terms of laboratory parameters (D-dimer, INR, hemoglobin, platelet count), no significant difference was observed between the groups (*P*= 0.074, *P*= 0.808, *P*= 0.058, *P*= 0.312, respectively).

**Table 1:** Characteristics of the Patients with PTE as the First or Recurrent PTE Event

**Variables (n, %)**	**Recurrent PTE (n=166)**	**First PTE** **(n=1286)**	***P*** ** Value**
Age (y)*	62.0 (16‒97)	61.8 (20‒100)	0.921
BMI (kg/m^2^)*	31.0 (19.6‒50.3)	28.5 (15.8‒57.8)	< 0.001
Gender			
Male	81 (48.8)	662 (51.5)	0.515
Female	85 (51.2)	624 (48.5)	
Presence of comorbid diseases	147 (88.6)	976 (75.9)	< 0.001
DM	64 (38.6)	278 (21.6)	< 0.001
Obesity	89 (53.6)	345 (26.8)	< 0.001
HT	77 (46.4)	361 (28.1)	< 0.001
CAD	32 (19.3)	232 (18.0)	0.697
CVD	2 (1.2)	43 (3.3)	0.135
COPD	22 (13.3)	190 (14.8)	0.601

DM, Diabetes mellitus; HT, Hypertension; CVD, Cerebrovascular diseases; COPD, Chronic obstructive lung diseases; CAD, Coronary artery diseases * The Mann-Whitney U test was used to compare these variables. Results were presented as median (min-max).

 VTE risk factors were compared between the two groups. Obesity, presence of hereditary risk factors for thrombophilia, HT and DM were found to be associated with an increased risk of recurrent PTE (*P*< 0.001, *P*< 0.001, *P*< 0.001, *P*< 0.001, respectively). When hereditary risk factors were analyzed one by one; factor V Leiden mutation, protein C deficiency and genetic changes specified under the name of other prothrombotic risk factors in our study (activated protein C resistance, factor XIII deficiency, plasminogen activator inhibitor-1 deficiency, GP2b3A mutation) were observed more frequently in the recurrent PTE group (Table 2). On the other hand, malignancy and immobilization were found to be significantly higher in patients in the first PTE group (*P*= 0.029, *P*= 0.014) (Table 2).

**Table 2:** Comparison of Groups in Terms of Predisposing Factors for Venous Thromboembolism

**Variables (n, %)**	**Recurrent PTE (n=166)**	**First PTE** **(n=1286)**	***P*** ** Value**
DM	64 (38.6)	278 (21.6)	< 0.001
Obesity	89 (53.6)	345 (26.8)	< 0.001
HT	77 (46.4)	361 (28.1)	< 0.001
CVD	2 (1.2)	43 (3.3)	0.135
Cancer	20 (12.0)	244 (19.0)	0.029
Surgery (arthroscopic, laparoscopic and others)	13 (7.8)	167 (13.0)	0.057
Immobilization	18 (10.8)	239 (18.6)	0.014
Major trauma	3 (1.8)	21 (1.6)	0.869
APS	2 (1.2)	5 (0.4)	0.154
Pregnancy/Post-partum period	0 (0.0)	15 (2.4)	0.149
OCT/HRT	1 (1.2)	21 (3.4)	0.275
Presence of hereditary risk factors for thrombophilia	22 (13.3)	66 (5.1)	< 0.001
Factor V Leiden mutation	8 (4.8)	18 (1.4)	0.002
Prothrombin G20210A	0 (0.0)	6 (0.5)	0.378
Deficiency of protein C	7 (4.2)	16 (1.2)	0.004
Deficiency of protein S	5 (3.0)	15 (1.2)	0.069
MTHFR mutation	5 (3.0)	23 (1.8)	0.281
Other hereditary risk factors	7 (4.2)	12 (0.9)	0.003

DM, Diabetes mellitus; HT, Hypertension; CVD, Cerebrovascular diseases; COPD, Chronic obstructive lung diseases; CAD, Coronary artery diseases; APS, antiphospholipid antibody syndrome; OCTS/HRT, Oral contraceptive treatment/Hormone replacement treatment; MTHFR, Methylene tetrahydrofolate-reductase.

 In the multivariable regression analysis, obesity was found to be the independent risk factor that increased the risk of recurrence the most. It was identified that the probability of recurrence after the first PTE attack in obese patients increased 4.13 times compared to the normal population (95% CI: 2.431‒7.042, *P*< 0.001). The presence of hereditary risk factors was also found to be the second risk factor that increased the risk of recurrence after obesity. It was observed that the risk of recurrence was increased 2.86 times in patients with hereditary risk factors compared to the normal population (95% CI: 1.646‒4.81, *P*< 0.001). While the presence of protein C deficiency and other hereditary risk factors, was observed to be statistically significant, factor V Leiden mutation did not reach statistical significance (OR: 1.804; 95% CI: 0.654‒4.976; *P*= 0.254). It was identified that the risk of recurrence increased 1.73 times in HT patients and 1.59 times in DM patients compared to the normal population (95% CI: 1.090‒2.322; *P*= 0.016, and 95% CI: 1.205‒2.505; *P*= 0.003, respectively).

 Cancer and immobilization, which were significantly more common in the first PTE group, did not reach significance in multivariable regression analysis (OR:0.723, 95% CI:0.435‒1.200, *P*= 0.209 - OR:0.595, 95% CI:0.353‒1.002, *P*= 0.051) (Table 3).

**Table 3:** Multivariable Regression Analysis for Pulmonary Thromboembolism Recurrence

**Risk factors**	**OR**	**95% CI**	***P*** ** Value**
BMI	0.990	0.902‒0.991	0.020
Immobilization	0.595	0.353‒1.002	0.051
DM	1.591	1.090‒2.322	0.016
Obesity	4.138	2.431‒7.042	< 0.001
HT	1.737	1.205‒2.505	0.003
Cancer	0.723	0.435‒1.200	0.209
Presence of hereditary risk factors for thrombophilia	2.865	1.646‒4.81	< 0.001
Factor V Leiden mutation	1.804	0.654‒4.976	0.254
Deficiency of protein C	2.964	1.074‒8.181	0.036
Other hereditary risk factors	4.239	1.439‒12.486	0.009

BMI, Body mass index; DM, Diabetes mellitus; HT, Hypertension.

**Figure 1:**
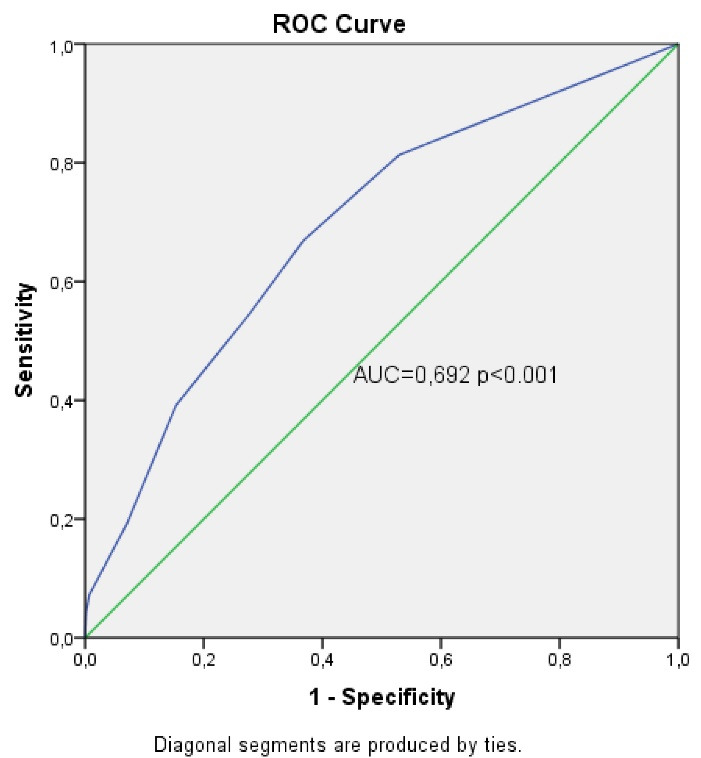
ROC Curve for the DOTH2 Scoring System

 A scoring system was created, called DOTH2, that can be used to predict the risk of recurrent PTE with variables that were statistically shown to increase the risk of recurrent PTE (Table 4). The score of each variable in the scoring system was created based on the odds ratios in the regression analysis. Accordingly, diabetes was scored as 1 point, HT as 1 point, presence of hereditary risk factors for thrombophilia as 2 points and obesity as 3 points. The capacity of the scoring system in predicting the risk of recurrence was analyzed using receiver operating characteristics (ROC) curve analysis (Figure 1). In the analysis, the area under the curve was found to be 0.692, and the 95% confidence interval was 0.650‒0.735. The significant cut-off value was 1.5 points. Based on the significant cut-off value, the sensitivity of our scoring system was 66.9%, the specificity was 63.2%, the positive predictive value was 19%, and the negative predictive value was 93.7%. The risk of recurrence in the patients identified as high-risk in the scoring system was 3.47 times higher than those identified as low-risk (95% CI: 2.435‒4.728). In our study, the 1-year recurrence rate was 2.1% in patients identified as low-risk ( < 2 points) during the first PTE attack. It was observed that 39% of these patients with recurrent PTE were cancer patients.

**Table 4:** DOTH2 Prediction Score for Pulmonary Thromboembolism

**Risk Predictor**	**Scoring**
D: DM	1 Point
O: Obesity	3 Points
T: Thrombophilia	2 Points
H: HT	1 Point

*Note:* Total score = 2, < 2 point = Low risk, ≥ 2 point = High risk DM, Diabetes mellitus; HT, Hypertension.

 When the relationship between the anticoagulant drugs used in the patients in the recurrent PTE group and the duration of recurrence was examined, no significant relationship was found between the drugs used and the duration of recurrence (*P*= 0.293).

## Discussion

 Identification of risk factors associated with recurrence in PTE has been the subject of many studies. In these studies, many different risk factors such as male gender, older age, obesity, lipid-lowering drugs, high hematocrit levels, high D-dimer levels, presence of antiphospholipid antibodies and hereditary risk factors for thrombophilia were associated with recurrence.^4-10^ However, an individual marker may fail to predict the risk of recurrence of PTE. Therefore, scoring systems would be more valuable and hence attractive as easy-to-use tools offering quantitative results. Consistent with the literature, obesity, presence of hereditary risk factors for thrombophilia, DM and HT have been found to increase the risk of recurrent PTE. Our scoring system, which we created based on these risk factors, also achieved a high negative predictive value in determining the risk of PTE recurrence.

 Studies in the literature suggest that PTE is more frequent in patients over 50 years of age, but there is no relationship between recurrence and gender and age.^4^ In our study, the mean age was found to be compatible with similar studies, and no statistically significant difference was observed between the groups in terms of age and gender.

 Obesity (BMI ≥ 30 kg/m^2^) was identified as a risk factor that significantly increases recurrent PTE. This result was consistent with other studies examining the relationship between obesity and recurrent PTE. In the study by Eichinger et al, recurrence was observed at 9.3% among patients with a BMI within the normal range ( < 25 kg/m^2^), 16.7% among patients who were overweight (25‒30 kg/m^2^) and 17.5% among patients who were obese ( > 30 kg/m^2^). It was also found that obesity increases the risk of recurrence by 1.6 times.^11^ In another study conducted in a population of women under 45 years of age, a BMI greater than 25 was found to be associated with higher risk of recurrence.^12^ It is known that both obesity and obesity-related comorbid diseases trigger thrombus formation by increasing procoagulant activity and decreasing mobilization. Since the effects of these factors that cause thrombus formation on the organs can continue for a long time, the risk of recurrence may be higher in these patients compared to the normal population in the long term, even if there is weight loss. Therefore, we think that controlling obesity in the early period is important in terms of preventing recurrence.

 In the literature, different results have been reported in studies that investigated the relationship between the hereditary risk factors and recurrent PTE. In a meta-analysis study by Ho et al, the risk of recurrent VTE increased 1.41 times (95% CI: 1.14‒1.75) in patients with factor V Leiden mutation, and 1.72 times (95% CI: 1.27‒2.31) in patients with prothrombin G20210A mutation.^13^ In addition, protein C, protein S, and antithrombin deficiency were associated with an increased risk of recurrence in a few studies.^14^ Contrary to these studies, in the study by Christiansen et al, it was shown that the risk of recurrence increased by 1.4 times in patients with hereditary risk factors compared to the normal population, but no relationship was found between the causes of thrombophilia and the increased risk of recurrence.^15^ It was shown in our study that the presence of hereditary risk factors for thrombophilia increased the risk of recurrence by 2.8 times. The risk of recurrence was observed to increase significantly in the presence of protein C deficiency and other prothrombotic risk factors in our study. The presence of factor V Leiden mutation was observed more frequently in patients in the recurrent PTE group, but it was not found to be statistically significant. These results are compatible with other studies in terms of the relationship between hereditary risk factors and recurrence. In the study by Christiansen et al, exclusion of patients over 70 years, those diagnosed with cancer, and long-term use of anticoagulants after the first attack, may explain the contrary results of this study.^15^ In many studies, including our study, different hereditary risk factors for thrombophilia were found to be associated with recurrence. The different genetic backgrounds and exposure to different environmental conditions of the patients included in the study can explain these different results. Therefore, our study may contribute to the literature in terms of evaluating the genetic mutation data of our country and the relationship between hereditary risk factors for thrombophilia and recurrent PTE.

 Although there are studies presenting various results on the relationship between cardiovascular diseases and relapse, there is no study in the literature addressing the relationship between HT and recurrent PTE.^4,16,17^ In addition, HT is among risk factors for the first VTE (OR < 2) in the ESC Guidelines for the diagnosis and management of acute pulmonary embolism.^2^ In our study, HT was one of the most frequently observed comorbid diseases in both groups and it was observed that it increased the risk of recurrence significantly. Normal vascular endothelium inhibits thrombus formation by secreting many vasodilators and cytokines that inhibit platelet activation. Damage to the vascular endothelium causes increased procoagulant activity and thrombus formation.^18^ If the systemic blood pressure cannot be kept within normal limits for a long time after the first attack, the resulting endothelial damage may trigger the thrombus formation mechanism and cause recurrent PTE. Therefore, keeping systemic blood pressure within normal limits in patients with a diagnosis of HT is very important to reduce the risk of recurrence.

 Previous studies suggest that the increase in thrombin production and in the concentration of procoagulant cell-derived microparticles in type 2 DM patients may play an important role in the frequency of VTE in these patients.^10^ Increased frequency of VTE and risk of recurrence in DM patients have been shown in many studies. In a study by Zhang et al, the risk of recurrence in patients whose blood glucose control was not achieved was 1.38 times higher than patients whose blood glucose control was achieved, and 1.34 times higher than patients without diabetes.^16^ In the study by Piazza et al, DM was associated with a significantly increased risk of recurrent DVT, but not recurrent pulmonary embolism.^19^ These results had shorter follow-up periods of patients compared to other studies. In our study, the presence of DM increased the risk of PTE recurrence by 1.5 times, which is consistent with similar studies. As in similar studies in the literature, our study also shows that DM is an important risk factor for VTE recurrence. We believe that achieving blood glucose regulation in these patients will prevent continuous endothelial damage, venous stasis and hypercoagulability, and reduce the risk of recurrence.

 The presence of active cancer is known to be an important risk factor for recurrent VTE.^20^ However, the presence of active cancer was not found to be associated with recurrent PTE in our study. We might justify this result with the fact that since our hospital is a chest diseases branch hospital, the majority of cancer patients are lung cancer patients, which is one of the cancers with the shortest expected survival.

 Researchers have proposed various scoring systems to determine and standardize the risk of recurrence after the first PTE attack. The HERDOO2 score and the DASH score are the most well-known of these scoring systems.^8,9^ Rodger et al followed up patients with unprovoked VTE who had received 5‒7 months of anticoagulant therapy in terms of the risk of recurrence and the need for extended therapy. According to their study, hyperpigmentation, edema or redness of either legs, D-dimer ≥ 250 μg/L while on anticoagulation, BMI ≥ 30 kg/m^2^; or age ≥ 65 years were found to be predictive parameters for recurrence in women (HERDOO2 score). Women with two or more of these risk factors had a significantly higher risk of relapse than those with 1 or 0 risk factors.^8,21^ The VTE recurrence risk of women who have low risk (risk factor ≤ 1) was found at 2.7% and the VTE recurrence risk of women who have high risk (risk factor > 12) was found at 10.2%. In the study by Tosetto et al, the parameters were examined which predicted VTE recurrence in unprovoked VTE patients who used anticoagulant therapy for at least 3 months and whose treatment was completed. There were significant differences between patients with and without recurrent VTE in terms of age, gender, use of hormonal therapy during the first episode of VTE, and D-dimer status after anticoagulation (the DASH score). The annual risk of recurrence was found to be 3.1% in patients with a DASH score of 1 and below.^9^ In our study, according to our scoring system consisting of obesity (3 points), hereditary risk factors for thrombophilia (2 points), HT (1 point) and diabetes mellitus (DM) (1 point), patients with a score of 2 or more were found to be at high risk. In addition, our scoring system, unlike the HERDOO2 score, can be used not only for women but also for both genders. According to our scoring system, the risk of recurrent PTE was found to be 3.47 higher in high-risk patients. Accordingly, the fact that the patient has only obesity or only the presence of hereditary risk factors increases the risk of recurrent PTE by 3.5 times. Because of this important risk difference, using the scoring system to separate the high and low risk groups is an important step in treatment and follow-up. In addition, it is very important to try to eliminate risk factors in patients with these risk factors and to administer treatments to protect them against risk factors.

 In addition, annual recurrence rates in patients assessed as low-risk by our scoring system were consistent with data from clinically used and validated scoring systems (2.1% annual recurrence rate). The high negative predictive value of our scoring system shows that it is a highly accurate method in detecting patients with low risk for relapse and that anticoagulant therapy can be safely terminated in these patients. In the HERDOO2 scoring system study, it was suggested that an annual rate of 3% or less for recurrent VTE would be required to terminate anticoagulant therapy.^8^ This supports the notion that the use of our scoring system will be beneficial in avoiding the costs and complications of long-term anticoagulant therapy. However, for use in daily practice, it needs to be validated in a similar population with another prospective study, such as the HERDOO2 score. Since the presence of cancer, which is accepted as a risk factor for recurrent PTE in the literature, was found to be inversely related to recurrent PTE in our study, we recommend that our scoring system should be applied in patients without active cancer. We believe that our scoring system will achieve higher diagnostic performance in a population without cancer patients.

 Our study has some limitations that should be mentioned. The first of these is that it is single-centered and retrospective. Secondly, the fact that our hospital is a branch hospital serving only chest diseases patients limited our case diversity. Our third limitation is the loss of data during data transfer due to the fact that the hereditary risk factors panel was studied in another center.

## Conclusion

 In conclusion, it should be noted that the risk of recurrence might be higher in patients with DM, HT, obesity and any of the hereditary risk factors compared to the normal population. In order to prevent recurrence, it is be important to manage comorbid diseases adequately in these patients and to devise an effective PTE treatment plan. We believe that using scoring systems to determine the risk of recurrence in acute PTE patients will be valuable and interesting as it is easy-to-use, gives quick results and provides quantitative results.
